# Correlation of early over-sedation with clinical outcomes in mechanically ventilated patients: further studies are needed

**DOI:** 10.1186/s13054-014-0597-7

**Published:** 2014-11-14

**Authors:** Shou Yin Jiang, Ying Ying Zhao, Xiao Gang Zhao

**Affiliations:** Department of Emergency Medicine, Second Affiliated Hospital, Zhejiang University School of Medicine, 88 Jiefang Road, Hangzhou, 310009 PR China

In a previous issue of *Critical Care* we read with great interest the article by Tanaka and colleagues [1] who studied the relationship between early deep sedation and clinical outcomes of mechanically ventilated patients in Brazilian ICUs. Accordingly, early deep sedation (defined as a Glasgow Coma Scale score of less than 9 with sedatives on the second day of mechanical ventilation) is a common characteristic in the ICU and was associated with higher disease severity, longer duration of ventilator support, and more tracheostomies. This study suggests that patients who are over-sedated during the early course of ventilation may suffer from deleterious outcomes. Though enlightening, it has raised more questions than it has answered. Most of all, the correlation between early over-sedation and clinical outcomes seems to be a bit far-fetched. According to Table 2 [1], the baseline data between light and deep sedation were not comparable, as admission Simplified Acute Physiology Score 3 and Sequential Organ Failure Assessment score on day 1 were significantly lower in light sedation than in deep sedation (both *P* =0.001). This implies that the disease severity in deep sedation was significantly higher than in light sedation. Thus, it is easy to consider that those worse clinical outcomes in deep sedation were related to the increased disease severity rather than the deepened sedation. Second, the authors should be cautious when interpreting the trends to increased mortality in deep sedation in circumstances of a nearly significant *P* value because such a description gives a misleading impression and does not mean that statistical significance can be shown by enlarging sample size [2].

## Authors’ response

Lilian Maria Sobreira Tanaka, Luciano Cesar Pontes Azeved, and Jorge Ibrain Figueira Salluh for the ERICC (Epidemiology of Respiratory Insufficiency in Critical Care) study investigators

We thank Zhao and colleagues for their interest in our study on the association of early sedation strategies with outcomes of adult critically ill patients under mechanical ventilation in a multicenter cohort study in Brazil. We would like to provide answers to their important concerns.

We did consider the possibility that deep sedation could be a ‘marker’ of more severe clinical conditions. So we performed a multivariate analysis, as shown in Table 4 [1]. We entered all variables that had significant or nearly significant *P* values or those that are considered clinically relevant regardless of the *P* value in the univariate analysis. Although the univariate analysis (depicted in Table 3 [1]) indicated an almost significantly higher hospital mortality (*P* =0.051) in the group under deep sedation, the multivariate analysis showed that it was independently associated with hospital mortality. Deep sedation not only was an independent predictor of outcome but also, of the five variables selected, presented the highest odds ratio (2.36) with a *P* value of 0.004 for deep sedation.

Therefore, we believe that our study highlights the profound clinical impact of deep sedation in the outcomes of critically ill patients under mechanical ventilation. As our data clearly demonstrate, such a factor is relevant even in the early phase of ICU admission. This is even more relevant as it is a potentially modifiable factor with the implementation of light sedation protocols.
